# Using an integrative machine learning approach utilising homology modelling to clinically interpret genetic variants: CACNA1F as an exemplar

**DOI:** 10.1038/s41431-020-0623-y

**Published:** 2020-04-20

**Authors:** Shalaw R. Sallah, Panagiotis I. Sergouniotis, Stephanie Barton, Simon Ramsden, Rachel L. Taylor, Amro Safadi, Mitra Kabir, Jamie M. Ellingford, Nick Lench, Simon C. Lovell, Graeme C. M. Black

**Affiliations:** 1 Division of Evolution and Genomic Sciences, School of Biological Sciences, Faculty of Biology, Medicines and Health, University of Manchester, Manchester Academic Health Science Centre, Manchester, UK; 2 Manchester Centre for Genomic Medicine, Central Manchester University Hospitals NHS Foundation Trust, Manchester Academic Health Sciences Centre, St Mary’s Hospital, Manchester, UK; 3 Congenica Ltd, Biodata Innovation Centre, Wellcome Genome Campus, Hinxton, Cambridge UK

**Keywords:** Genetic databases, Genetic testing

## Abstract

Advances in DNA sequencing technologies have revolutionised rare disease diagnostics and have led to a dramatic increase in the volume of available genomic data. A key challenge that needs to be overcome to realise the full potential of these technologies is that of precisely predicting the effect of genetic variants on molecular and organismal phenotypes. Notably, despite recent progress, there is still a lack of robust in silico tools that accurately assign clinical significance to variants. Genetic alterations in the *CACNA1F* gene are the commonest cause of X-linked incomplete Congenital Stationary Night Blindness (iCSNB), a condition associated with non-progressive visual impairment. We combined genetic and homology modelling data to produce CACNA1F-vp, an in silico model that differentiates disease-implicated from benign missense *CACNA1F* changes. CACNA1F-vp predicts variant effects on the structure of the *CACNA1F* encoded protein (a calcium channel) using parameters based upon changes in amino acid properties; these include size, charge, hydrophobicity, and position. The model produces an overall score for each variant that can be used to predict its pathogenicity. CACNA1F-vp outperformed four other tools in identifying disease-implicated variants (area under receiver operating characteristic and precision recall curves = 0.84; Matthews correlation coefficient = 0.52) using a tenfold cross-validation technique. We consider this protein-specific model to be a robust stand-alone diagnostic classifier that could be replicated in other proteins and could enable precise and timely diagnosis.

## Introduction

Over the past decade, high-throughput DNA sequencing technologies have revolutionised the management of individuals with rare genetic disorders, enabling timely and precise diagnosis, and facilitating personalised medicine approaches [1]. For genetically heterogenous conditions such as hereditary hearing loss and inherited retinal disorders (IRDs), genomic testing has been shown to have significant clinical utility, leading to improved management [2]. In these conditions, variant detection can provide a molecular diagnosis in over 50% of patients [3, 4]. However, distinguishing the disease-causing variants from the many potentially functional variants present in any human genome remains particularly challenging [5].

IRDs is a heterogeneous group of disorders that affect ~1 in 3000 people [6] and are a leading cause of blindness in working age adults in the UK [7]. Congenital stationary night blindness (CSNB; also known as congenital stationary synaptic dysfunction/disorder) is a non-progressive form of childhood-onset IRD that is associated with variable combinations of night vision problems, reduced visual acuity, myopia, and nystagmus. X-linked incomplete CSNB (iCSNB; also known as type 2 CSNB (OMIM 300071)) is the most prevalent CSNB subtype and it is classically caused by variants in the *CACNA1F* gene [8]*. CACNA1F* (Gene ID 300110) consists of 48 exons (ENST00000376265.2) and encodes a polypeptide (1977 amino acids) that forms the pore of a voltage-gated calcium channel, Ca_v_1.4 α1 [9, 10]. *CACNA1F* (NM_005183.3). Its function involves sustaining continuous calcium dependent glutamate release from the photoreceptors to bipolar cells in the retina [11]. Over fifty *CACNA1F* missense variants are described to cause iCSNB on the human gene mutation database (HGMD v2019.4) [12] the majority of which are functionally uncharacterised. Improved prediction of the likely phenotypic consequences of missense variants in *CACNA1F* is therefore key to the molecular diagnosis of patients with iCSNB.

A number of in silico tools can be used for interpreting the effects of sequence variants, both in research and in clinical laboratory settings. Four commonly used tools include Sorting Intolerant From Tolerant (SIFT, [13]) which uses a sequence homology-based method, polymorphism phenotyping v2 (PolyPhen2, [14]) which utilises a sequence and structure-based approach, combined annotation dependent depletion (CADD, [15]) which uses a supervised learning method, and consensus deleteriousness score (CONDEL, [16]) which uses a consensus-based approach [17]. The latest version of CONDEL combines the predictions of two other tools, mutation assessor [18] and the Functional Analysis Through Hidden Markov Models ([19]) using weighted averaging.

Despite relative success in differentiating between disease-causing and benign variants in some genes, these tools are not consistently effective in their predictions [20]. Even in combination, their efficiency has been shown to be gene-dependent [21]. Here, we integrate detailed genetic *CACNA1F* data with homology modelling of the Ca_v_1.4 α1 protein structure. We show, using CACNA1F-variant predictor (CACNA1F-vp), that protein-specific structural analysis has the ability to improve performance in differentiating disease-implicated missense changes from other potentially functional variants.

## Methods

### Datasets

The HGMD^R^ database was used to retrieve missense variants that have been previously associated with clinical phenotypes (*n* = 63; database accessed October 2017). The ClinVar database [22] was also interrogated and a literature search, using the search term ‘CACNA1F AND mutation*’, was carried out at the same time; no further changes were identified. DNA changes associated with disease in patients tested at the Manchester Genomic Diagnostic Laboratory (MGDL), a United Kingdom Accreditation Service Clinical Pathology Accredited medical laboratory (Clinical Pathology Accredited identifier, no. 4015) were also included (*n* = 9; database accessed October 2017). The guidelines set out by the American College of Medical Genetics and Genomics and Association of Molecular Pathology [23] were used to evaluate the latter set of variants. The Genome Aggregation Database (gnomAD, accessed October 2017) [24] was used to identify a set of presumably benign, “control” variants (hereafter referred to as benign variants). All missense changes detected in males were selected (*n* = 322). According to the gnomAD curation team every effort was made to exclude individuals with severe paediatric diseases from the dataset so we do not expect the overwhelming majority of these variants to be associated with iCSNB [24] [online: http://gnomad.broadinstitute.org/faq; accessed January 2019].

### Homology modelling

A homology model of Ca_v_1.4 α1 was generated using MODELLER v9.17 [25], since its 3D structure has not been experimentally determined. The *CACNA1F* sequence from UniProt (Uniprot ID: O60840, [26]) was used to identify the structure of the rabbit Ca_v_1.1 complex from the Protein Data Bank (PDB, [27]) as a homologous structure (PDB ID: 5GJV). The sequences were aligned using Clustal Omega v1.2.3 [28] with default parameters. Approximately 64% sequence identity in the modelled regions suggests similarity in structure. Five models of Ca_v_1.4 α1 were built and the one with the lowest Discrete Optimised Protein Energy score was selected. PyMol [29] was used to visualise the model.

### Hypotheses and analyses

To examine van der Waals interactions in the model, hydrogen atoms were added with Reduce [30], and atomic contacts were calculated with Probe [31]. Amino-acid replacements were modelled and visualised using KiNG v2.23 [32]. All low energy side chain conformations (rotamers) were examined and the one with smallest van der Waals overlaps chosen for each variant. In addition, the Richards volume scale [33] was used to calculate the differences in residue volumes. A reference set of all possible differences in volume of all 20 amino acids was calculated; the results of this were divided into four bins based on volume change upon amino-acid replacement: <−42Å^3^ group, −42 to 0Å^3^ group, 0 to 42Å^3^ group and >42Å^3^ group. Similarly, both the disease-implicated and the presumably benign set of variants were divided into four bins and were later compared with a replication the same four bins of variants with the only difference of having the mutant/introduced residues randomly generated using Monte Carlo simulation to identify statistically significant differences (*p* < 0.05).

The EGS hydrophobicity scale [34] was used to calculate change in hydrophobicity arising from an amino-acid replacement. To investigate changes in charged residues, hydrophobic residues, and differences in spatial distribution of the variants between the two sets, a reference set of uniformly distributed variants that were randomly generated using Monte Carlo simulation was used.

*CACNA1F* orthologues (*n* = 23) were identified from UniProt and aligned using Clustal Omega with default parameters. A conservation score was calculated through generating a profile [35] where the alignment is converted into a position-specific scoring system. The frequency with which the residues occur at each position is scored and later used to measure conservation using the substitution matrix BLOSUM62 [36]. The intracellular and extracellular sides of the plain in the model were determined by defining the spatial distribution of the residues using angle calculations. The angle formed between three residues (the Cα atom of the query residues, the centre of mass of the protein, and the Cα of Lys383 chosen by inspection) was calculated. A residue was counted to be on the intracellular side of the plain if this angle was <90˚, otherwise on the extracellular side of the plain. The carboxyl terminal domain (CTD) and the unmodelled parts were excluded from this calculation.

The scripts used in this study are available on the GitHub repository (https://github.com/shalawsallah/CACNA1F-variants-analysis).

### Formulating pathogenicity criteria and evaluating prediction performance

We defined two datasets. Dataset D represents disease-implicated variants from HGMD^R^ and MGDL and dataset N represents presumably benign variants that were found in hemizygous state in the gnomAD cohort. We analysed these two datasets to find features that correlate with disease causality. The logistic regression algorithm “Logistic” [37] from the WEKA (Waikato Environment for Knowledge Analysis) machine learning package [38] was used with default parameters (weka.classifiers.functions.Logistic -R 1.0E-8 -M -1 -num-decimal-places 4) to classify the variants. Prior to this, the “ClassBalancer” and “Discretize” filters (weka.filters.MultiFilter -F “weka.filters.supervised.instance.ClassBalancer -num-intervals 10” -F “weka.filters.supervised.attribute.Discretize -R first-last -precision 6”) were applied successively to reweight the imbalanced classes in the data and increase performance, respectively.

The performance of this classifier was later compared to that of the other four classifiers described above using the area under the curve (AUC) of receiver operating characteristic (ROC, [39]). The AUC under the precision recall (PR) curve was also used to measure their performances in correctly identifying the true positives, i.e. disease-associated variants, among the true positives and false positives [40]. The Matthews correlation coefficient test (MCC, [41]) was used to measure the correlation between the actual class of variants and the predictions made by the classifiers. The Bonferroni correction was applied to correct for possible error rates in multiple comparisons, such as comparisons made in domains of the protein [42].

## Results

### *CACNA1F* variants identification

We identified 72 disease-implicated missense variants (dataset D) that were present in either HGMD^R^ (*n* = 63) or the MGDL database (*n* = 9). Next, we identified 322 presumably benign missense variants (dataset N) from gnomAD (the combined 394 variants are shown in Online Resource 1). Class assignment to datasets D and N was not definitive. Rather, it was assumed that the two groups of variants represented populations that were significantly skewed towards carrying disease-causing and benign variants, respectively.

### Performance of in silico tools

We then assessed the ability of four in silico tools to predict the class of the *CACNA1F* missense variants (Table 1). The performance of these tools was highly variable, with a notable variation in the false positive rate (FPR). However, when using the unscaled/raw scores of CADD, at a threshold of 5.25, instead of the recommended scaled scores, we found it to perform better (e.g. MCC = 0.53, up from 0.12, AUC ROC = 0.83, up from 0.79, and AUC PR = 0.44, up from 0.43). Furthermore, we found that changing the CONDEL-defined threshold from 0.52 to 0.65, i.e. a threshold specific to *CACNA1F*, results in a higher overall performance (e.g. MCC = 0.52, up from 0.17).

**Table 1:** The comparison of the true positive (TP) and false positive (FP) predictions of *CACNA1F* variants using four different tools (total positives and negatives = 72 and 322, respectively (NM_005183.3; ENST00000376265.2)); *FPR*: false positive rate.

Tools	Optimal threshold	TP	FP	FPR (%)
SIFT	0.05	65	171	53
PolyPhen2	0.85	62	132	41
CADD	15	70	277	86
CONDEL	0.52	69	255	79

### Homology modelling

**Fig. 1:**
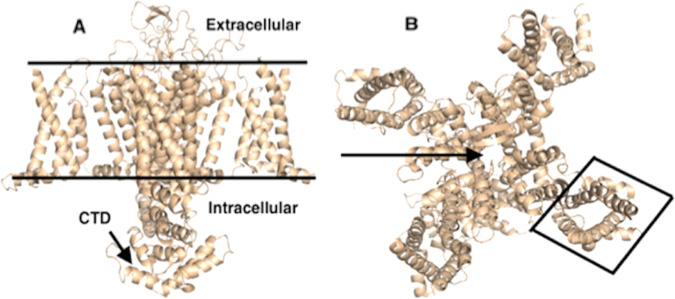
Model representation of the template structure (PDB ID 5GJV) used in homology modelling. The structure is of the mammalian voltage-gated calcium channel Ca_v_1.1 complex at a resolution of 3.6 angstroms [43]. The transmembrane domain (approximately within the lines) in the side view representation (**a**). The pore (indicated by an arrow) and the first four out of six segments (highlighted in the rectangle) of each of the four domains in top view representation (**b**).

In order to analyse the structural and physicochemical properties of missense variants, we generated a homology model of Ca_v_1.4 α1 and mapped the variants onto it. Approximately 2/3 of the human Ca_v_1.4 α1 protein could be modelled, i.e. residues 67–414, 516–766, and 858–1580. The parts that were not modelled had no homologous sequence in the template protein. Both termini of template Ca_v_1.4 α1 are on the cytoplasmic side and the structure has four (I–IV) transmembrane domains, each of which consists of six transmembrane α-helical segments (S1–S6). The fourth transmembrane helix (S4) is a voltage sensor, with S5 and S6 segments of each domain making the ion channel and selectivity filter (Fig. 1, [43]).

### Structural analysis as a means of assessing variant pathogenicity

In order to integrate clinical *CACNA1F* data with homology modelling of Ca_v_1.4 α1 we defined a set of structure-based parameters and determined their ability to differentiate variants from the D and N groups.

(i) *The Ca*_*v*_*1.4 α1 model*. Four regions of the human Ca_v_1.4 α1 sequence, residues 1–66, 415–515, 767–857, and 1581–1977, have no homologous residues in the rabbit Ca_v_1.1 protein used as a template for modelling. The majority of the variants, i.e. 68/72 (94%), from dataset D were found to be on regions shared by both the model and the template structure, i.e. modelled regions, (*p* < 0.0001) compared with only 200/322 (62%) of the variants from dataset N (*p* > 0.9). The regions absent from the model, i.e. unmodelled regions, contain only a small proportion of the variants from dataset D and are poorly conserved across ten human paralogues [43]. These data therefore suggest that variants found within the unmodelled regions are less likely to be disease-associated.

(ii) *Variant location within the Ca*_*v*_*1.4 α1 protein*. Visual inspection suggested that the majority of the variants from dataset D were found closer to the intracellular region than to the extracellular one. We therefore defined a plain through the centre of mass of the molecule and determined whether variants were on the extracellular or intracellular side of this plain. This defined 745 residues to be in the extracellular side of the plain and 621 in the intracellular side of the plain. The locations of the variants differed between the two groups, with group D variants more frequently seen on the intracellular side of the plain of domain I (*p* = 0.048 (a significant *p* value must be <0.005 following Bonferroni correction)).

(iii) *Conservation of mutated residues*. Of the 72 mutated residues from dataset D, 69 were conserved among the 24 species considered (i.e. had a calculated conservation score ≤5 out of 10). In contrast, of the 322 mutated residues from dataset N only 177 were conserved and 145 non-conserved (i.e. had a conservation score of ≥ 6 out of 10 (*p* = 1.2 × 10^−11^, Mann–Whitney U test)).

**Fig. 2:**
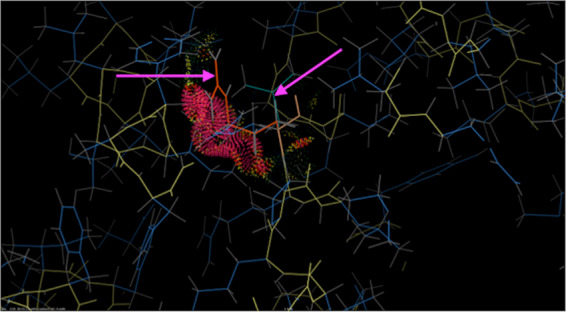
Protein structure modelling for a disease-implicated variant. In testing the molecular goodness-of-fit for the disease-implicated *CACNA1F* (NM_005183.3; ENST00000376265.2) variant c.647 T > G p.(Leu216Arg), the red spikes reflect an overlap of van der Waals interaction between the surrounding residues and the introduced arginine (in orange) in place of the mutated leucine (in green) highlighted by the arrows.

When considering only the modelled regions, 67 of the 68 mutated residues from dataset D were established as conserved compared with 158 of the 200 mutated residues from dataset N (*p* = 2.36 × 10^−25^, Mann–Whitney U test). It can therefore be concluded that changes in conserved residues, the majority of which are on the modelled regions, are shown to be more likely disease-associated.

(iv) *Changes in residue-volume and molecular goodness-of-fit test*. Replacement of amino-acids may result in steric clashes with neighbouring regions of the protein model. To determine whether this is the case we assessed all low-energy side chain conformations [44] and evaluated their “goodness-of-fit” using the Probe software [31]. There is a significant difference between the two sets of data (*p* = 0.001, Mann–Whitney U test) with a higher number of variants in dataset D having a more negative Probe score compared with the group N variants (*p* = 0.03 at Probe scores <180, Mann–Whitney U test). This indicates the introduction of van der Waals overlaps resulting in steric clashes (Fig. 2).

The above finding is in accordance with the differences found in volume-change between the two groups of variants (*p* = 0.03, Mann–Whitney U test) with a higher number of variants in dataset D resulting in the replacement of smaller amino-acids with larger ones (i.e. a size change <−42Å^3^, *n* = 23/72 for dataset D, *n* = 59/322 for dataset N; *p* = 0.046, Mann–Whitney U test). This difference was also observed when changes in volume in group D variants were compared with the reference set of volume changes (*p* = 0.04, Mann–Whitney U test), in contrast to comparing the group N variants to the reference set (*p* = 0.50, Mann–Whitney U test). Therefore, the changes that lead to disruption of packing, and a more negative Probe score are more likely to be disease-associated.

(v) *Changes in charged residues*. Cav1.4 α1 is a voltage-gated calcium channel and alterations in charge are likely to affect its function. The replacement of neutral or negatively charged residues with positively charged residues, (gain of positive charge), was found to be more frequent among variants from dataset D, *n* = 17/72, than dataset N, *n* = 34/322, throughout Cav1.4 α1 (*p* = 0.036). There was more frequent replacement of positively charged residues with neutral or negatively charged residues (loss of positive charge) amongst variants from dataset D than dataset N in the fourth transmembrane helix (S4) (i.e. the voltage sensor) of all domains combined (*p* = 0.002), *n* = 4/6, and in S0-4 of domains II (*p* = 0.01(significant *p* value must be <0.005 following Bonferroni correction)), *n* = 2/4, and in S0-4 of domain IV (*p* = 0.002 (significant *p* value must be <0.005 following Bonferroni correction)), *n* = 3/5.

(vi) *Changes in hydrophobic residues*. Since Cav1.4 α1 is a transmembrane protein, variants involving replacement of hydrophobic residues were considered. The replacement of hydrophobic residues among variants from dataset D in domain I, *n* = 12/16, is correlated with pathogenicity (*p* = 0.015 (a significant *p* value must be <0.005 following Bonferroni correction)).

### The pathogenicity criteria

The pathogenicity criteria identified were used as features (Online Resource 1) by a logistic classifier to differentiate between the disease-implicated and presumably benign datasets as a composite assessment:

- Variants in sequences shared by the template structure and the model
- Loss of positively charged residues in the fourth transmembrane helix (S4) of the four homologous domains
- Gain of positively charged residues throughout the protein
- Loss of hydrophobic residues in domain I
- Variants at conserved residues
- Variants found in the lower half of domain I
- Variants resulting in the introduction of larger residues in place of smaller residues
- A more negative goodness-of-fit, i.e. Probe, score

### Machine learning application

The logistic regression model “Logistic” from WEKA was used to classify the variants. The performance of the binary classifier is evaluated using a ROC curve [45] which measures trade-offs between the sensitivity and the specificity of the classifier at different thresholds. An optimum threshold can allow for a higher true positive rate (TPR) or a lower FPR, as required, or a combination of these in a diagnostic classifier. To account for the imbalance between the two classes in the data however, the ROC curve is combined with a PR curve to evaluate the true positives among the overall positive predictions.

**Fig. 3:**
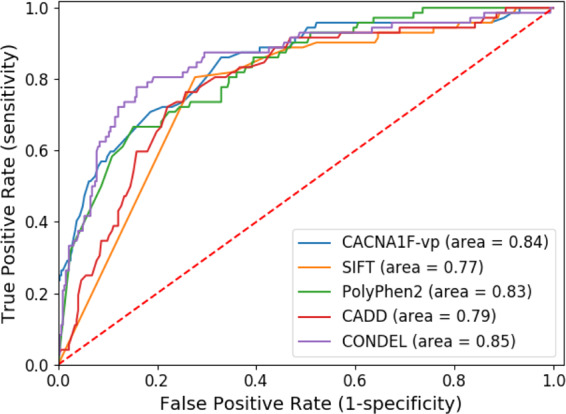
ROC curves for the different classifiers. The predictive power of the protein-specific (CACNA1F-vp) model is comparable to that of the four tools, using 72 disease-implicated and 322 presumably benign *CACNA1F* variants, shown by an area under the receiver operating characteristic (ROC) curve of 0.84.

**Fig. 4:**
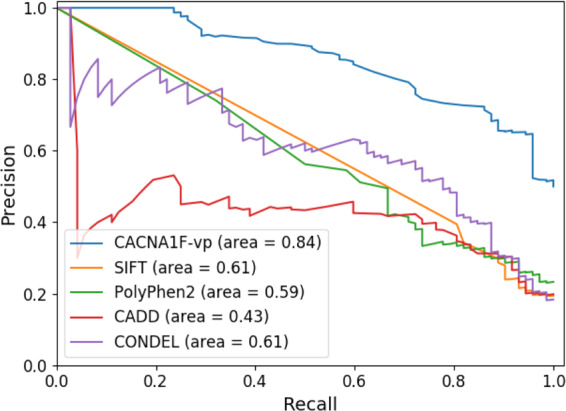
PR curves for the different classifiers. The precision of the protein-specific (CACNA1F-vp) model is outperforming that of the four tools, using 72 disease-implicated and 322 presumably benign *CACNA1F* variants, shown by an area under the precision recall (PR) curve of 0.84.

The logistic model performance was compared with four commonly used in silico prediction tools (Table 2). Its performance in differentiating between the two classes of variants (AUC ROC = 0.84) is comparable to that of the other four classifiers (Fig. 3). Notably, the larger area under the logistic model PR curve (AUC PR = 0.84) represents a comparably high precision (representing a low FPR), and a high recall or sensitivity, i.e. a low false negative rate (Fig. 4).

**Table 2:** Comparing the predictions and the overall performance of the different tools shows a high recall rate at the expense of the precision rate at optimum thresholds for all the tools except for CACNA1F-vp. CACNA1F-vp has also a higher MCC score (MCC scores range from 1 to −1 with 1 being a perfect correlation between predictions and the classes, and −1 being an inverse correlation); total disease-implicated and benign variants = 72 and 322, respectively (NM_005183.3; ENST00000376265.2); *AUC ROC*: area under the receiver operating characteristic curve, *AUC PR*: area under the precision recall curve, *TPR*: true positive rate, *FPR*: false positive rate, *PPV*: positive predictive value, *MCC*: Matthews Correlation Coefficient.

Tools	Threshold	Recall/TPR (%)	FPR (%)	Precision/PPV (%)	AUC ROC	AUC PR	MCC
SIFT	0.05	88	53	28	0.77	0.61	0.3
PolyPhen2	0.85	86	41	32	0.83	0.59	0.35
CADD	15	97	86	20	0.79	0.43	0.12
CONDEL	0.522	96	79	21	0.85	0.61	0.17
CACNA1F-vp	0.567	86	33	72	0.84	0.84	0.52

## Discussion

The interpretation of the large number of genetic variants generated through current gene sequencing techniques poses a significant challenge [46]. Computational prediction tools go some way to address this major issue but have been shown to frequently be inconsistent [20]. In this study, we produced a *CACNA1F*-specific variant classifier through analysing sequence and structural data of the protein and its variants. This protein-specific approach was used as an alternative to currently available tools that tend to be less intuitive and often perform in a contradictory fashion [47, 48]. Our analysis was enabled through the use of a 3D homology model of the protein structure that allowed structural analysis of 94% of the disease-implicated and 62% of the presumably benign variants. Clearly such analysis may not be possible for proteins where there is limited knowledge of the protein structure. Notably, the modelled region contained the majority of disease-implicated variants and appeared to be conserved among the orthologs of Cav1.4 α1. In contrast, the regions that were not included in the model contain only a small proportion of the disease-implicated variants and are poorly conserved across the orthologs and ten human paralogues of Cav1.4 α1. These results indicate a strong correlation firstly between the modelled protein regions and pathogenicity, and secondly between conservation and pathogenicity for this molecule.

The loss of positively charged residues in the fourth transmembrane helix (S4) found in the disease-implicated variant set, is thought to cause disturbance in voltage-dependence functionality [49]. The outward movement of gating charges in S4 seem to result in bending of S6 and opening the pore [50]. Positively charged S4 residues form salt bridges with the negatively charged residues on S1-3 [50, 51]; it can be speculated that altering these interactions affects channel structure and function. In contrast, disease-implicated variants leading to gain of positively charged residues may affect the overall function through interference with calcium ion selectivity and permeability [49]. In the CTD, such alterations may also interfere with inhibition of calcium dependent inactivation. Notably, this important regulatory functional domain tends to be less tolerant to variation [52].

We compared the prediction performances of four in silico tools to that of CACNA1F-vp, and found that the presented protein-specific model was specific and accurate. It could differentiate between disease-associated and benign variants as well as the other in silico tools (AUC ROC = 0.84; Table 2). Furthermore, our predictive model outperforms the other tools in correctly classifying the majority of the true disease-associated variants with a lower false positive prediction (AUC PR = 0.84; Table 2). Including more presumably benign than disease-implicated variants in the analyses, i.e. having an imbalanced dataset, could improve the ROC curve without any real improvement in sensitivity or specificity. Evaluation using a PR curve is immune to this effect of an imbalanced dataset. This makes the PR curve a more robust measure to evaluate the specificity of each tool. CACNA1F-vp misclassified seven disease-implicated variants. These include the four disease-implicated variants found outside of the modelled regions of the protein (c.1301 C > T p.(Ala434Val), c.1464 G > T p.(Glu488Asp), c.2390 A > T p.(Glu797Val), and c.2542 G > A p.(Gly848Ser)), and one variant (c.1903G > A p.(Val635Ile)) that was seen in the gnomAD population at a high frequency (320/150041 alleles). The homology model was less informative for the variants found outside of the modelled regions. Hence, the lack of structural information about these variants may be a strong factor in their misclassification. CACNA1F-vp also misclassified 125/322 (39%) benign variants (Online Resource 2). A recent study found that disease-implicated *CACNA1F* variants are present in gnomAD, which might be due to overlooked/undiagnosed cases in this dataset [53]. When we used a more stringent criterion to define benign variants (presence in the gnomAD dataset in hemizygous state in at least five individuals) we found that the misclassification rate of CACNA1F-vp was lower (16/52; 30%). Overall, we found significant differences between the CACNA1F-vp predictions and those of SIFT, CADD, and CONDEL (*p* < 0.00001, McNemar chi square test [54]).

We found that adjusting the variant-pathogenicity thresholds defined by CADD and CONDEL improves the performance of these tools (MCC increases from 0.12 & 0.17 to 0.53 & 0.52, respectively). Therefore, a protein-specific pathogenicity-threshold in these tools further validates the advantage of using a protein-specific approach. A factor that could inflate the performance of these in silico methods is that the data used in testing these tools (in the present study) may have been utilised initially to train them. However probable, this was difficult to confirm.

Important insights could be gained by comparing the characteristics of the presumed disease-associated (HGMD^R^, MGDL) and the presumed benign (gnomAD) variants. One of the key differences was in the molecular “goodness-of-fit” test where the deleterious packing interactions were shown to be greater amongst disease-implicated variants and are likely to lead to structural instability and functional abnormality. Intriguingly, a small number of the presumably benign variants (*n* = 19/322) were also found to have significantly disordered packing interactions (Online Resource 3); the majority of these changes (14/19) were among the CACNA1F-vp misclassified variants (Online Resource 2). A possible explanation for this is the inaccuracies in the homology modelling process around these missense changes. Alternatively, it is not implausible that some of these benign variants are in fact disease-associated (especially the extremely rare ones such as c.2221 C > T p.(Leu741Phe) which is found in 1/86168 gnomAD alleles), Importantly, gnomAD is population rather than an unaffected control database and some individuals may in fact have iCSNB.

This study has important limitations. First, the missense changes that fall outside of the reliably modelled regions are more difficult to interpret using the approach outlined. This is highlighted by the misclassification of the four disease-implicated variants that fall outside of the modelled regions. Second, the protein-specific nature of our classifier has significant advantages but limits the number of available variants to train the model. Given the prevalence and degree of allelic heterogeneity of iCSNB, assembling a large independent variant dataset to test the classifier’s performance was not possible. Third, we did not take into account factors like penetrance and expressivity in assembling the disease-implicated (dataset D) and presumably benign (dataset N) variant datasets. It is worth noting though that, despite the fact that a number of different ophthalmic conditions has been linked to *CACNA1F* variants (including iCSNB, Åland eye disease and X-linked cone-rod dystrophy 3), incomplete penetrance is certainly not a frequent feature of *CACNA1F*-related disorders [52, 55]. Intriguingly, eight disease-implicated variants were also present in gnomAD in hemizygous state (and where therefore also included in the presumably benign dataset; Online Resource 4). This highlights the fact that certain variants would have been incorrectly annotated; clearly, this lack of definitive variant class assignment negatively affects the performance of CACNA1F-vp.

We can conclude that CACNA1F-vp can form the basis of an effective test. Its relatively higher precision compared with existing tools may help pinpoint disease-associated variants among background variation, facilitating the process of diagnosing patients with iCSNB. Importantly, wrongly diagnosing affected individuals can cause distress to the patient and their family and can lead to further unnecessary investigations (for example repeated electrodiagnostic assessments). Obtaining co-segregation and functional data is undeniably important and necessary but this information is often difficult or impractical to get. We therefore believe that the presented classifier has a role in the evaluation of individuals with iCSNB. Finally, it can be speculated that through studying different molecules using similar approaches, a set of pathogenicity rules will emerge including protein-specific, family-specific or even perhaps more general rules.

## Supplementary information

Online Resource 1

Online Resource 2

Online Resource 3

Online Resource 4
